# P08-02 Critical elements of sports programs for socially vulnerable adults: a concept mapping study

**DOI:** 10.1093/eurpub/ckac095.115

**Published:** 2022-08-29

**Authors:** Güven Alarslan, Dico de Jager, Kirsten Verkooijen

**Affiliations:** 1 Health and Society, Wageningen University and Research, Wageningen, The Netherlands; 2 Health and Society, Wageningen University and Research, Utrecht, The Netherlands

**Keywords:** sports programs, critical elements, socially vulnerable adults, positive outcomes

## Abstract

**Background:**

Sports programs are recognised as a promising way to contribute to the personal development of socially vulnerable adults. However, it remains unknown which elements of sports programs are critical for social inclusion and personal development to occur. Knowledge on these critical elements is needed to improve sports programs and to maximize positive outcomes.

**Methods:**

Data were collected and analyzed using Concept Mapping (CM), which is a standardized systematic tool to visualize relations between different concepts by collecting and sorting ideas in groups and ranking them in terms of importance (1-5 Likert-scale). A total of 14 sports coaches, 5 program coordinators, 8 social workers and 5 advisors partook in our CM study, making up four groups of informants. Data among participants of the sports programs have been collected in a separate study.

**Results:**

Altogether, sports coaches provided 152 elements, program coordinators provided 81 elements, social workers provided 115 elements, and the advisors provided 95 elements that they deemed critical for positive outcomes of sports programs serving socially vulnerable adults. Both overlapping and unique elements were provided. The role of the sports coach had the highest average importance score (>4.00) and was equally important for all four groups. Elements related to facilities of the sports activity, such as accessible and diverse activities appeared to be more important for social workers and advisors than for program coordinators and sports coaches. In fact, elements related to facilities of the sports program had the lowest importance score among program coordinators and sports coaches. Elements related to external partners involved in the sport program (e.g., clear agreements with partners, continuous funding) showed to be important to program coordinators and advisors, but less important to the sports coaches. Social workers did not mention elements related to external partners. All four groups deemed elements related to the personal development of the participant, such as working towards a goal and intrinsic motivation important.

**Conclusions:**

Training the sports coaches properly and keeping involved care workers in close contact enables participants to achieve better positive outcomes. Sports programs should be experienced as a safe and positive learning environment participants should be encouraged to work on their personal goals without forcing them. For the continuity of a sports program, collaboration with municipalities and social care organizations are essential.

